# Occurrence, Removal and Bioaccumulation of Perfluoroalkyl Substances in Lake Chaohu, China

**DOI:** 10.3390/ijerph16101692

**Published:** 2019-05-14

**Authors:** Xu Pan, Jing Ye, Hui Zhang, Jun Tang, Dandan Pan

**Affiliations:** 1 College of Resources and Environment, Anhui Agricultural University, Key Laboratory of Agri-Food Safety of Anhui Province, Hefei 230036, China; xupan2341@163.com (X.P.); m13606800364@163.com (J.Y.); tangjun@ahau.edu.cn (J.T.); 2 Environmental Protection Monitoring Station of Chaohu Authority, Hefei 230071, China; jddyahau@126.com

**Keywords:** perfluorinated compounds, Lake Chaohu, occurrence, distribution, bioaccumulation

## Abstract

The perfluoroalkyl substances (PFAAs) have received growing attention in recent years as emerging contaminants in the aquatic environment. The occurrence, removal and bioaccumulation of fourteen PFAAs (C4–C14 carboxylate; C4, C6, C8 sulfonates) were investigated in Lake Chaohu, China. The concentrations of the selected PFAAs in inflowing river, lake water and sewage treatment plant (STP) samples were analyzed by ultra performance liquid chromatograph (UPLC–MS/MS). The results showed that perfluorohexanesulfonic acid (PFHxS), perfluorobutane sulfonate (PFBS), perfluorobutyric acid (PFBA) and perfluoropentanoic acid (PFPeA), instead of perfluorooctanoic acid (PFOA), were predominant PFAAs in the inflowing river and lake water with maximum concentrations in the ranges of 52.2–1866 and 27–236 ng L^−1^, respectively. The highest concentrations of total PFAAs were detected in the western rivers. The effluents from seven STPs were likely important sources of PFAAs in surface water, and the amount of the daily fluxes in the effluent were 132 g for short-chain PFAAs and 109 g for long-chain PFAAs. PFAAs were widely detected in Chinese icefish (*Neosalanx tangkahkeii taihuensis*) collected from Lake Chaohu, with maximal concentrations ranging from 1.79 ng g^−1^ to 50.9 ng g^−1^. The logarithmic bioaccumulation factors of perfluorodecanoic acid (PFDA, 3.5), perfluorooctane sulfonate (PFOS, 3.35) and perfluorononanoic acid (PFNA, 3.31) indicated the bioaccumulation of these long-chain PFAAs. This study is useful for enhancing our understanding of the pollution profiles of PFAAs and their environmental health risk in the freshwater lake.

## 1. Introduction

Perfluoroalkyl substances (PFAAs) are organofluorine compounds in which all the hydrogen atoms on the carbon chain are replaced by fluorine atoms [1]. These PFAAs include C4–C12 perfluorinated carboxylates (PFCAs), perfluorobutane sulfonate (PFBS), perfluorohexanesulfonic acid (PFHxS) and perfluorooctane sulfonate (PFOS), among which C4–C6 PFCAs and PFBS belong to short-chain PFAAs, while C7–C12 PFCAs, PFHxS and PFOS belong to long-chain PFAAs. Due to their physicochemical properties, such as oil and water repellency and resistance to heat or chemical reactions, PFAAs are widely used as protectants for textiles, stain repellents, personal care products, pesticides, fire-fighting uniforms and emulsifying agents [2]. PFAAs are persistent in the environment because of their strong carbon-fluorine covalent bond. As a result, PFAAs are ubiquitous in the environmental media, including purified drinking water, surface water, groundwater, air, sludge, soil, sediments, outdoor and indoor dust, biota, and polar ice caps [3,4,5,6,7,8]. Field studies have shown that PFAAs can bioaccumulate and biomagnify through food webs [9,10,11]. Previous researches have revealed the adverse effects of PFOS and perfluorooctanoic acid (PFOA) on humans and aquatic organisms, such as neonatal mortality and carcinogenicity, inhibitory effects on *Chlorella vulgaris* and endocrine disrupting effects in male rare minnow [12,13,14]. The persistence, bioaccumulative properties and potential toxicity of PFAAs have raised considerable concern globally. PFOS and its salts are currently listed in persistent organic pollutants (POPs) under the Stockholm Convention.

Sewage treatment plants (STPs) are considered as a significant source of PFAAs to aquatic environments [15,16,17]. The influence of STP effluents on receiving rivers has been documented in China, Germany, Canada, Spain and Japan [18,19,20,21,22,23]. There are many studies investigating the occurrence, transport and fate of PFAAs in surface waters all over the world [24]. Bioaccumulation and biomagnification in aquatic food webs have also been reported in many studies [25,26,27]. However, a paucity of studies has focused on a multimedium analysis of PFAAs in a complex and open catchment, where wastewater, surface water and biota from rivers and lakes should be considered together.

Lake Chaohu is the fifth largest freshwater lake in China, which serves as a drinking water resource, irrigation water, aquaculture farms and a recreational attraction. With the rapid economic growth and urban development during the last several decades, the lake has been seriously polluted by wastewater and waste from industry, agriculture and daily life. Many studies have shown that Lake Chaohu is a significant sink for organic pollutants [28,29,30,31,32]. A previous study, conducted in 2011, reported the occurrence of PFAAs in Lake Chaohu, where long-chain PFAAs (e.g., PFOA) was the predominant PFAA in the surface water [33]. However, the regulations on PFOS, PFOA and related compounds result in a sharp increase in demand for other PFAAs in the market. This shift in production and application could be imaged by the contamination profiles and trends of PFAAs in the environment [34,35]. For instance, PFHxS, rather than PFOS or PFOA, was predominant in the surface water in Taihu Lake in recent years [36]. It should be noted that PFHxS has been added to the candidate list of substances of very high concern by the European Chemicals Agency and fit the requirements of Annex D of the Stockholm Convention [37,38]. Thus, it is important to explore whether the changes of the contamination profile observed in Taihu Lake will occur in other regions of the world. Given that Lake Chaohu is one of the most heavily polluted lakes in China, investigation on the change of the pollution profile and trend for PFAAs in this lake will provide a comprehensive understanding of the risks of PFAAs to the aquatic environment. Such an investigation is also imperative for the proper pollution control strategy on all PFAAs. In addition, the mass load of PFAAs from the domestic effluent has not previously been estimated. Therefore, the objectives of this study are as follows: (1) To investigate the occurrence of short- and long-chain PFAAs in Lake Chaohu and its inflowing rivers, (2) to estimate the daily flux of PFAAs from STPs, (3) and to examine the bioaccumulation of PFAAs in the dominant fish species *Neosalanx tangkahkeii taihuensis* in Lake Chaohu.

## 2. Materials and Methods

### 2.1. Chemicals and Standards

Fourteen PFAAs were selected as target chemicals, including three short-chain PFCAs of perfluorobutyric acid (PFBA, C_4_HF_7_O_2_), perfluoropentanoic acid (PFPeA, C_5_HF_9_O_2_) and perfluorohexanoic acid (PFHxA, C_6_HF_11_O_2_), eight long-chain PFCAs of perfluoroheptanoic acid (PFHpA, C_7_HF_13_O_2_), PFOA (C_8_HF_15_O_2_), perfluorononanoic acid (PFNA, C_9_HF_17_O_2_), perfluorodecanoic acid (PFDA, C_10_HF_19_O_2_), perfluoroundecanoic acid (PFUnDA, C_11_HF_21_O_2_), perfluorododecanoic acid (PFDoDA, C_12_HF_23_O_2_), perfluorotridecanoic acid (PFTriDA, C_13_HF_25_O_2_) and perfluorotetradecanoic acid (PFTDA, C_14_HF_27_O_2_), one short-chain perfluoroalkyl sulfonic acid (PFSA) of PFBS (C_4_F_9_O_3_S) and two long-chain PFSAs of PFHxS (C_6_F_13_O_3_S) and PFOA (C_8_H_17_O_3_FS). Perfluoro-n-(1,2,3,4-^13^C_4_) butanoic acid (^13^C_4_-PFHeA), perfluoro-n-(1,2,3,4-^13^C_4_)octanoic acid (^13^C_4_-PFOA), Perfluoro-n-(1,2,3,4,5-^13^C_5_)nonanoic acid (^13^C_5_-PFNA), perfluoro-n-(1,2-^13^C_2_)decanoic acid(^13^C_2_-PFDA), perfluoro-n-(1,2-^13^C_2_) dodecanoic acid (^13^C_2_-PFDoDA), perfluoro-n-(1,2-^13^C_2_)undecanoic acid (^13^C_2_-PFUnDA), sodium perfluoro-1-hexane(^18^O_2_) sulfonate (^18^O_2_-PFHxS) and sodium perfluoro-1-(1,2,3,4-^13^C_4_) octanesulfonate (^13^C_4_-PFOS), were used as internal standards. All of the analytical standards (purity ≥ 95%) were purchased from J.T. Baker(Phillipsburg, NJ, USA). Ammonium acetate (purity ≥ 98%) and methanol (HPLC grade) were obtained from TEDIA (Fairfield, OH, USA).

### 2.2. Sample Collection

**Figure 1:**
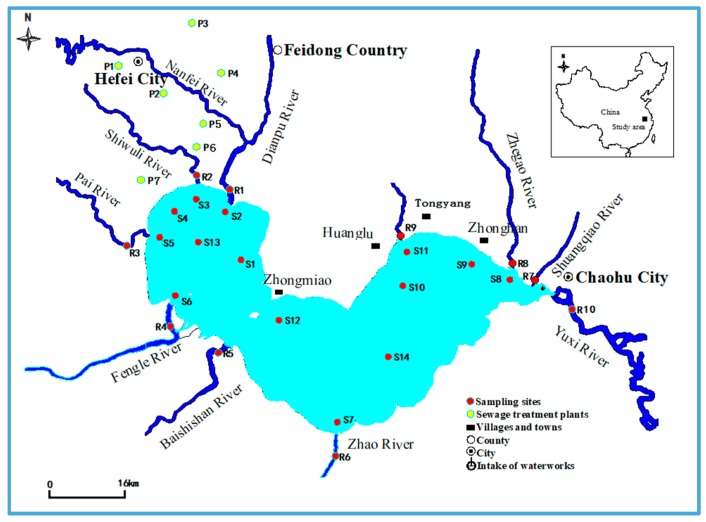
Sampling sites in Lake Chaohu.

Water and unsexed fish samples were collected in November 2015. The detailed locality of the sampling sites is shown in Figure 1. Polypropylene (PP) bottles were used to collect surface water samples at 24 locations in the main inflowing rivers (R1 to R10, *n* = 30) and the lake (S1 to S14, *n* = 42). Grab samples of influent and effluent water from seven sewage treatment plants (STP1 to STP7, *n* = 42) were collected to evaluate the mass loads of PFAAs from major STPs in Hefei City. Furthermore, fish (*Neosalanx tangkahkeii taihuensis, n* = 24) samples were collected from a fishing boat in Lake Chaohu to assess the potential levels of PFAAs to fish. This fish species is one of the dominant and main economic species of Lake Chaohu [39]. In addition, this species is a predator in the food web of Lake Chaohu. Thus, it is a good indication of PFAA bioaccumulation in other aquatic species. The species identification was confirmed by the analysis of morphological traits according to the description in the Atlas of freshwater fish in China [40]. Fish treatment was approved by the Institutional Animal Care and Use Committee of Anhui Agricultural University. Before analysis, water samples were vacuum filtered with PP fiber filters (0.45 µm, Whatman, Clifton, NJ, USA) and stored at −50 °C. Each fish sample (composed of several individuals) were chopped and homogenized in a food processor (XIBEILE SQ2119N, Shanghai, China) to create a representative sample.

### 2.3. Sample Preparation and Analysis

River water, lake water and wastewater samples were extracted in triplicate according to previous methods [23,41]. Briefly, 500 mL of water sample spiked with 5 ng of internal standards was extracted by a solid phase extraction (SPE) method on Oasis^®^ WAX cartridges (500 mg/6 cc, Waters, Milford, MA, USA). The cartridges were preconditioned with 1% ammonium hydroxide in methanol and followed by pure water. A flow rate of 5 mL min^−1^ was maintained through the cartridges. The cartridges were then washed with pure water and were dried completely under vacuum. Afterwards, the target compounds were eluted in 4 mL of methanol and 4 mL of 1% ammonium hydroxide in methanol into a PP tube. The eluent was concentrated to approximately 20 µL under a gentle nitrogen stream and reconstituted using 1 mL of the sample diluents (50% methanol in water).

Fish samples were extracted and cleaned up according to a method previously reported by He and Chen [42] with some modification. An aliquot of 1.0 g (wet weight) of the fish sample was placed in a 15 mL PP tube containing 10 mL of acetonitrile with 0.1 mL of formic acid and 2 ng internal standards. The sample was vortexed for 2 min and then sonicated in an ultrasonic water bath for 20 min and centrifuged at 4500 rpm for 10 min. The supernatant was transferred to 50 mL PP centrifuge tubes. The sample was further extracted in the same step twice and the three supernatants were combined and then concentrated to 10 mL under a gentle nitrogen stream. Pure water (90 mL) was added to each concentrated sample, and the mixture was vortex-mixed. The mixture was cleaned up by the same method with the water samples and diluted with 1 mL of 50% methanol in water before analysis.

PFAAs were analyzed by an ultra-liquid chromatography-tandem mass spectrometer (UPLC-MS/MS). The UPLC-MS/MS method was modified from a previous study [23]. Chromatographic separation was performed on a 4.6 × 150 mm Waters BEH C18 column. The injection volume was 5 μL and the mobile phases were 5 mM ammonium acetate solution (A) and methanol (B), at a flow rate of 0.3 mL min^−1^. The gradient elution started with a 0.2 min isocratic step at 10% of solvent B, then was ramped to 90% in 0.5 min, held at 95% for 1 min and brought back to 90% at 6.5 min. Quantification of PFAAs was performed on a triple quadrupole mass spectrometer (Waters TQs) with electrospray negative ionization in multiple reaction monitoring (MRM) modes. Two MRM transitions were used to identify all the target PFAAs compounds (except for PFBA and ^13^C_4_-PFBA), while one transition was chosen for the quantification of target compounds. For PFBA and PFHxA (as they can only provide one MRM transition), the confirmation was performed based on one MRM transition in addition to the retention time. The instrumental parameters were optimized to reach the maximum response (Table S1).

### 2.4. Quality Assurance/Quality Control (QA/QC)

A sample blank was routinely included in each batch of samples to eliminate any external source of contamination. Method blanks, prepared with 0.5 L Milli-Q water according to the same sample pretreatment procedures, were used to assess potential contamination during sample extraction, cleanup and analysis. In addition, an isolator column (2.1 × 50 mm, 5 μm, Waters) was equipped between the autosampler and the pump to eliminate the potential effect of polyfluoroalkyl substances on the solvent or pipe system.

Matrix-spike recoveries of individual PFAAs were obtained by spiking the 14 target analytes (5 ng for each compound) into randomly collected surface water, influent water, effluent water and fish samples. The recovery tests were performed in quintuplicate, with maximum relative standard deviations (RSDs) of less than 16%. Recoveries of PFAAs (Table S2) spiked into samples ranged from 58–107% for surface water, 56–120% for influent water, 52–106% for effluent water and 64–109% for fish samples. The limits of quantitation (LOQs) of PFAAs were estimated to be 0.01–0.04 ng L^−1^ in surface water, 0.04–0.6 ng L^−1^ in influent water, 0.02–0.30 ng L^−1^ in effluent water and 0.05–0.5 ng g^−1^ (wet weight) in fish samples, respectively. The final concentrations of PFAAs reported in this study were not recovery corrected.

## 3. Results and Discussion

### 3.1. Occurrence, Removal and Flux of PFAAs from STPs

**Figure 2:**
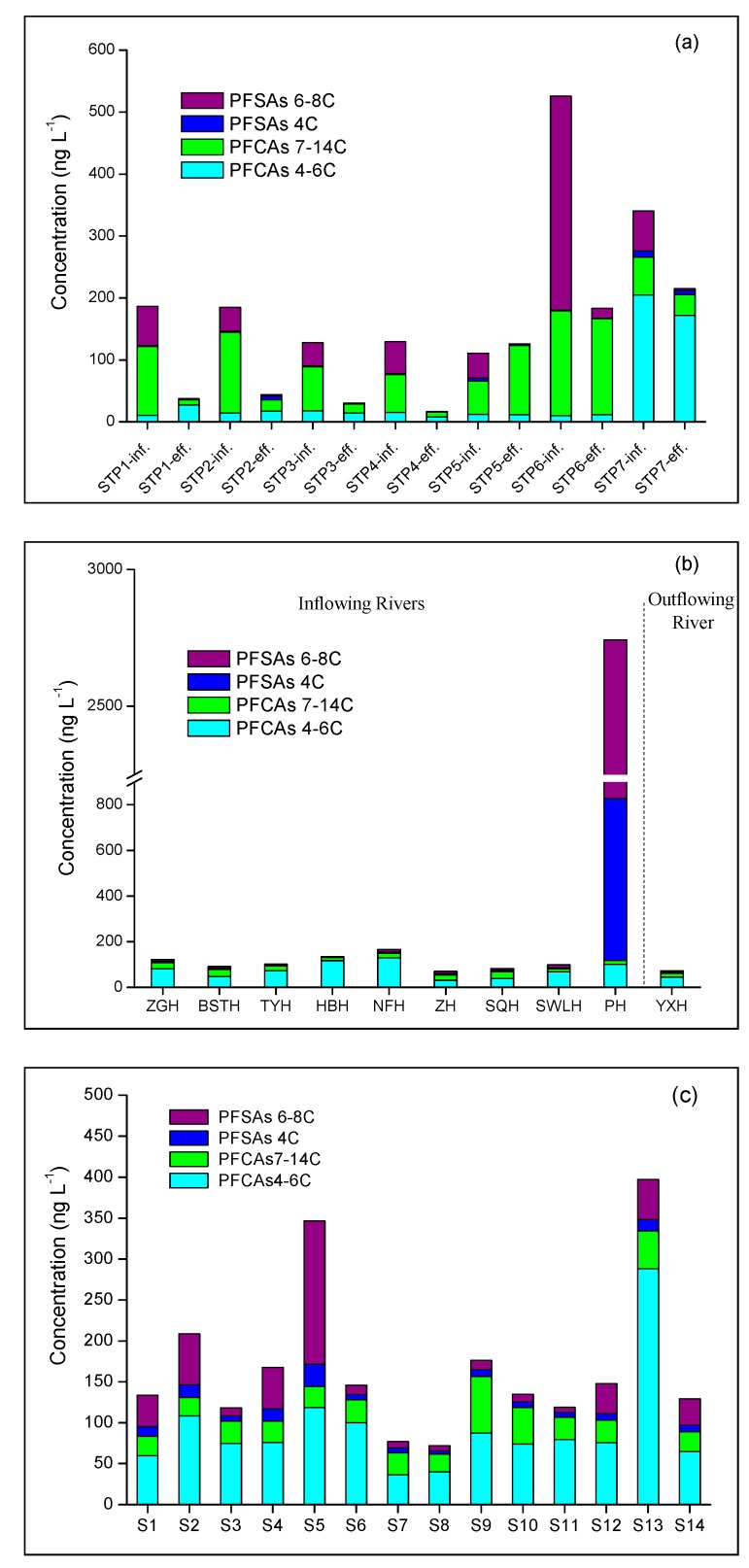
Cumulated perfluorinated carboxylates (PFCAs), perfluoroalkyl sulfonic acid (PFSAs) and concentration according to the short (4–6 C PFCAs and 4 C PFSA) and long (7–14 C PFCAs and 6–8 C PFSAs) length of the chain present at different sampling sites in (**a**) sewerage treatment plants, (**b**) rivers and (**c**) Lake Chaohu.

STPs play an important role in the protection of the aquatic environment around the city cycle. Influents of STPs are likely to receive PFAAs from industrial and household sources [7]. However, PFAAs could not be completely removed by STPs. As a result, effluents of STPs become important point sources of PFAAs to the aquatic environment [16,19,22,23]. The concentration of 14 PFAAs presents in influent and effluent water samples from seven STPs in Hefei city are shown in Figure 2a according to the short (4–6 C PFCAs and 4 C PFSA) and long (7–14 C PFCAs and 6–8 C PFSAs) length of the chain. The range of total concentrations of PFAAs (ΣPFAAs) is 111–526 ng L^−1^ and 17–215 ng L^−1^ in influent water and effluent water, respectively. Among the detected PFAAs, PFOS (C8), which belongs to long-chain PFSAs, was the predominant PFAA in influent water with a maximal concentration of 333 ng L^−1^ found in STP6 followed by PFOA (C8) with a maximal concentration of 163 ng L^−1^ found in STP6. For all influent samples, the total concentrations of short-chain PFAAs (ΣPFAAs) in STP1 to STP6 ranged from 10.8 to 19 ng L^−1^ and were obviously lower than long-chain ΣPFAAs (94 to 515 ng L^−1^), while short-chain ΣPFAAs (214 ng L^−1^) displayed higher levels than long-chain ΣPFAAs (126 ng L^−1^) in STP7. This finding may be attributed to the larger use of short-chain PFAAs in the area of STP7, which is near the car manufacture industry and chemical industry area. According to the concentration of PFAAs in effluent samples, the long-chain PFAAs were obviously eliminated in STP1, STP2, STP3, STP4 and STP6, with removal rates ranging from 67% to 95%. Furthermore, PFOS was not detected (or the concentration was below the LOQ) in the effluents of STP1, STP2, STP3, STP4 and STP5, while concentrations ranged from 27.3 to 57.7 ng L^−1^ in influent water. Similar results were reported by Zhang et al. [23], and the sorption onto the primary sludge or grid residue rather than biodegradation are considered to be an explanation for the removal rates. The higher removal efficiency of long-chain PFAAs is likely due to their higher adsorption ability to sludge compared to short-chain PFAAs, which can be explained by the increased *K*_oc_ values with increasing carbon chain length [43].

The daily mass flux of PFAAs (g/d) in Lake Chaohu from effluents in STPs was calculated using the following equation: Mass flux (g/d) = the concentrations of PFAAs (ng L^−1^) in effluents × the daily discharge (m^3^/d). As shown in Table 1, the sum of daily fluxes in the effluent was 0.6–107 g for short-chain PFAAs and 0.88–67.1 g for long-chain PFAAs. The total flux of PFAAs was 241 g in the main STPs in Hefei city. STP7 was the dominant source with total fluxes up to 129 g, due to their higher treatment capacity and effluent concentrations. In contrast, STP4 only contributed to a small amount of PFAA discharge, with the total flux below 2 g.

It should be noted that our results were obtained based on a single time point from the influent and effluent. In fact, wastewaters are highly variable in the concentration of pollutants. Thus, the sampling uncertainty existed in our study, which may lead to overinterpretation of measured data. Ort et al. have demonstrated that the magnitude of sampling uncertainty depends mainly on the number of pollutant peaks and the sampling frequency [44]. Correspondingly, they proposed a condensed step-by-step sampling guide in wastewater systems, which can largely reduce the sampling uncertainty [45]. Therefore, further work on the fate of PFAAs in wastewater systems should select an appropriate sampling mode and frequency according to this sampling guide.

### 3.2. Occurrence and Distribution of PFAAs in The Rivers around Lake Chaohu

Of the 14 target compounds, 11 PFAAs were frequently detected in the water samples from the inflowing rivers at the ng L^−1^ level (Table 2). Long-chain PFAAs were less common in water samples than short-chain ones except for PFHxS, and PFUnDA (C11), PFDoDA (C12), PFTriDA (C13) and PFTDA (C14) were not detected. PFHxS (C6) and PFBS (C4) were the predominant PFAAs in the river samples with maximal concentrations of 1866 and 710 ng L^−1^, respectively. The concentrations of short-chain PFCAs (4–6 C) and PFSA (4 C) and long-chain (7–14 C) PFCAs and PFSAs (4–6 C) in inflowing rivers were 32.6–101 ng L^−1^, 11.5–29.7 ng L^−1^ 2.44–2576 ng L^−1^ and 1.87–49.4 ng L^−1^, respectively (Figure 2b). Previous reports verify that the short-chain PFAAs experience limited sorption to soil or are less retarded by sediment compared to long-chain PFAAs in groundwater [46,47]. Therefore, the ubiquitous presence of short-chain PFAAs in rivers may pose a threat to the safety of the groundwater supply in the Lake Chaohu basin.

Regarding the spatial distributions of the PFAAs among the inflowing rivers, the total concentrations of PFAAs in western rivers (Paihe River, Nanfei River and Hangbu River) were generally higher than those in eastern rivers (Zhegao River, Tongyang River and Shuangqiao River) and southern rivers (Baishishan River and Zhaohe River). The highest pollution level was observed in the Paihe River with a maximum total concentration of 2743 ng L^−1^, which was ten times higher than that in other rivers. As the unique outflowing river, Yuxihe River exhibited a lower pollution level than all inflowing rivers with a total concentration of PFAAs of 73.5 ng L^−1^. The western rivers flow through Hefei City, which is the largest city around Chaohu Lake. It should be noted that the level of ΣPFAAs of the Shiwuli River is comparable to that of southern rivers. This observation was inconsistent with the results of previous studies, which suggested that high levels of polybrominated diphenyl ethers [48] and antibiotics [29] were found in the Shiwuli River. These contradictory results may be due to the ecological restoration project carried out in the Shiwuli River by the government of Hefei city before our sampling campaign. The moderate pollution level of PFAAs in eastern rivers could be explained by the input of effluents from STPs in Chaohu city. The southern rivers run through rural areas with less pollution emission. Thus, our results suggest that human activities play a key role in the distribution of PFAAs in Lake Chaohu.

### 3.3. Occurrence and Distribution of PFAAs in Lake Chaohu

All 14 selected PFAAs were widely detected in Lake Chaohu (Table 2) with frequencies higher than 70%. Consistent with the results of the inflowing rivers, the short-chain PFAAs generally displayed higher levels than long-chain PFAAs (Figure 2c). For short-chain PFAAs, relatively higher concentrations were found for PFPeA (9.06–236 ng L^−1^), PFBA (12.7–46.2 ng L^−1^) and PFBS (4.39–27 ng L^−1^). With regard to the long-chain PFAAs, high levels were found for PFHxS (3.44–168 ng L^−1^) and PFOA (17.1–33.28 ng L^−1^).

The detailed concentrations of 14 PFAAs from S1 to S14 are shown in Table S3. Generally, the western half of the lake had higher concentrations than the eastern half, with a maximum concentration of 397 ng L^−1^ at S13. The source of the Paihe River (2742 ng L^−1^ in river water) resulted in a higher concentration of PFAAs (420 ng L^−1^) in the estuary site (S5) compared to the Zhaohe River estuary site (82.1 ng L^−1^ at S7), suggesting a greater contribution of input of PFAAs by cities compared to rural areas. Furthermore, the PFAAs at sites of villages and towns near the North Lake (S9–S12) clearly showed the effects of industrial activities on Lake Chaohu.

### 3.4. Composition of PFAAs in Lake Chaohu and Global Comparison

**Figure 3:**
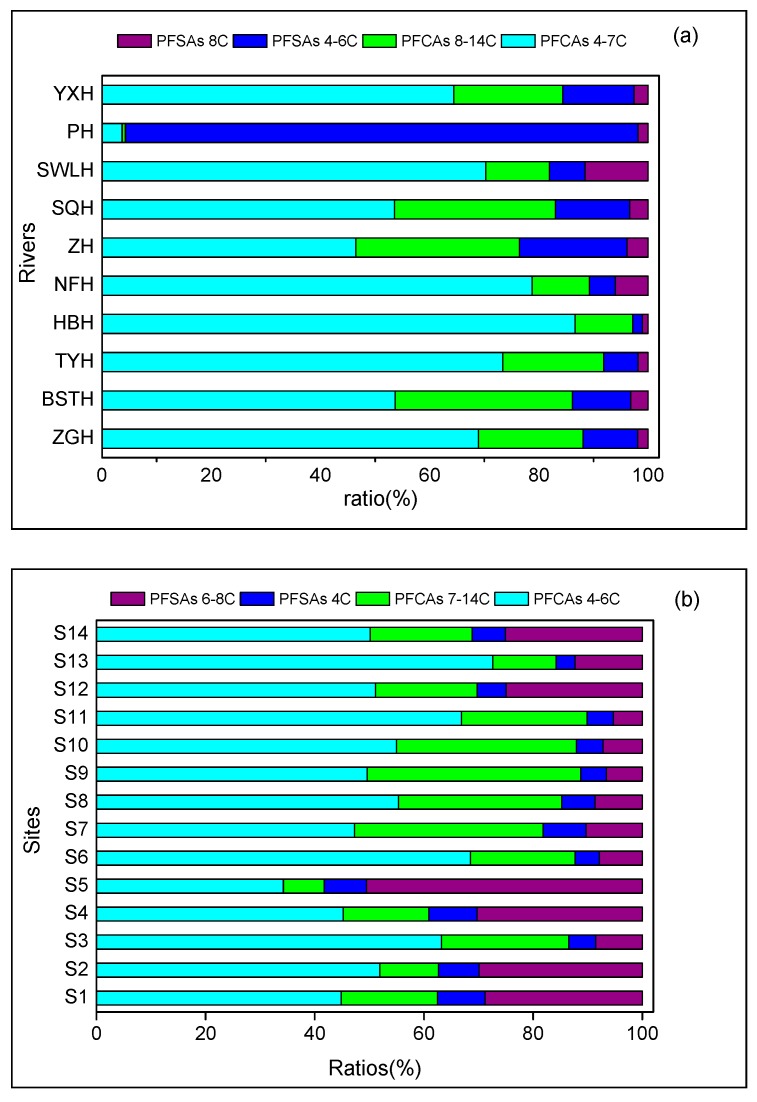
Composition of PFAAs according to the short (4–6 C PFCAs and 4 C PFSA) and long (7–14 C PFCAs and 6–8 C PFSAs) length of the chain present at different sampling sites in (**a**) rivers and (**b**) Lake Chaohu.

The profile of PFAAs in rivers and the lake is illustrated in Figure 3, and the detailed descriptions are shown in Tables S4 and S5. In all inflowing rivers except Paihe River, short chain PFCAs accounted for the highest proportions (46–87%) followed by long-chain PFCAs (11–32%), short-chain PFSAs (2–20%) and long-chain PFSAs (1–12%). For the Paihe River, 94% of ΣPFAAs were contributed by short-chain PFSAs, suggesting that the profile composition of PFAA sources in this area is different from other areas. Similar to river waters, the lake sites were also predominated by short-chain PFCAs, with the exception of S5, where the highest proportions were found for short-chain PFSAs (50.53%). The high contribution of short-chain PFSAs at S5 could be observed because this site is near the outlet of the Paihe River. Considerable changes in the composition of PFAAs in Lake Chaohu were observed when our results were compared with those from earlier research, conducted in 2011 [33]. The proportion of the major long-chain PFAAs (PFOA, 23–78%) was always notably higher than the total proportion of the two major short-chains PFAAs (PFBA, 3–47% and PFHxA, 0–40%) in water samples collected in 2011 [33]. These results indicate that short-chain PFAAs and PFHxS have become the predominant PFAAs in Lake Chaohu during 2011–2015. The increase of short-chain PFAAs and PFHxS in Lake Chaohu might be the result of recent production and use of PFHxS and short-chain PFAAs (e.g., PFBS) as alternatives, due to regulations on PFOS and PFOA. This trend was in line with two recent studies conducted by Ma [36] and Cui [49], who found that PFHxS replaced PFOA and PFOS as the predominant PFAAs in Taihu Lake from 2014 to 2015 and in Baiyangdian Lake from 2008 to 2018.

In addition to the changes in the composition of PFAAs in Lake Chaohu, the levels of almost all target compounds were elevated in comparison with those sampled in 2011 [33]. In particular, the maximum concentrations of PFHxS and PFPeA in water samples were one or two orders of magnitude higher than those detected in 2011 [33]. Interestingly, higher concentrations of PFOA and PFOS were observed in water compared with those reported by Liu et al. [33]. This phenomenon might reflect the increasing use of PFOA and PFOS around Lake Chaohu, although they have been gradually replaced by PFHxS and short-chain PFAAs. All the 14 PFAAs have been reported to be prevalent at detectable concentrations in surface waters in other regions of the world (Table 3). PFOA and PFOS are the main PFAA components detected in many studied regions [50,51,52]. The concentrations of PFOA in the present study are higher than those in Swedish rivers [53], but lower than those previously reported in Taihu Lake [50] and the Bohai sea [52], as well as much lower than those in rivers in Japan [51]. The levels of PFOS in Lake Chaohu were comparable to those in Taihu Lake [50], the Bohai Sea [52] and Swedish rivers [53], but lower than those in Japanese rivers [51]. As the main PFAAs in Lake Chaohu, PFHxS and the short-chain PFPeA and PFBA were clearly higher than those in the surface waters in China and other countries [50,51,52,53]. The concentrations of other PFAAs, such as PFHxA, PFHpA and PFBS, were generally on the same order of magnitude as those in Taihu Lake [50], the Bohai Sea [52] and Swedish rivers [53], but considerably lower than those in rivers in Japan [51].

### 3.5. Occurrence of The Selected PFAAs in Biota Samples

The concentrations of PFAAs in fish samples are shown in Table 4.

All target PFAAs were detected in fish samples. PFOS, PFOA, PFBA, PFPeA, PFDA, PFUnDA and PFDoDA were detected in all the samples, while PFTriDA (92%), PFNA (85%) and PFTDA (85%) were found in the majority of the samples. PFHxA, PFHpA, PFBS and PFHxS were less frequently detected (<55%). PFOS (0.44–50.6 ng g^−1^) was found to be the dominant PFAA in fish samples. This observation is consistent with several previous studies, which found that PFOS was predominant in marine and freshwater fish samples from China, Greece and US [8,11,49,56,57]. For PFCAs in the present study, PFBA (C4, 1.42–19.3 ng g^−1^), PFDA (C10, 0.33–13.9 ng g^−1^), PFPeA (C5, 0.64–12.4 ng g^−1^) and PFUnDA (C11, 1.09–10.1 ng·g^−1^) were the four dominant PFCAs detected in fish. The predominance of C10 and C11 PFCAs is similar to that reported for fish samples previously collected from Lakes Chaohu and Taihu [8,11]. Given the low bioaccumulation potential of short-chain PFAAs in biota [58], the high concentrations of C4 and C5 PFAAs in this study could be due to their higher levels in lake water. PFHxS was also a dominant PFAA in lake water, but it was found in fish at notably low levels with maximal concentrations of 1.7 ng·g^−1^, indicating the low bioaccumulation potential of this compound in fish.

Bioaccumulation is a process in which chemical substances are absorbed in organisms via multiple exposure pathways including water and food [59]. The bioaccumulative potential of a compound can be expressed by the bioaccumulation factor (BAF), which is calculated as the ratio of the concentration of the chemical in biological tissue to that in water. Thus, BAF is an important indicator of the cumulative trend of pollutants in organisms. Chemicals are considered to be bioaccumulative when the BAF is greater than 1000, 2000 or 5000 L/kg by various regulatory authorities [60]. Based on the concentrations of PFAAs measured in water and the whole body of fish from Lake Chaohu, the BAFs of target PFAAs were calculated for fish from Lake Chaohu (Table 4). The greatest average log BAF for fish species was found for PFDA (3.50) followed by PFOS (3.35) and PFNA (3.31), whereas other PFAAs showed relatively lower average log BAF values, ranging from 1.45 to 2.85. This result agrees with the conclusions drawn by previous studies that PFCAs with ≤7 fluorinated carbons are not considered bioaccumulative, and the bioaccumulative potential is limited by the molecular size of PFCAs with ≥11–12 fluorinated carbons [8,11,26,49].

## 4. Conclusions

The present study showed that the target PFAAs were widely distributed in rivers, surface waters, wastewater and biota samples in Lake Chaohu. The prevalence of PFAA effluents of seven STPs demonstrated that traditional secondary treatment processes were not sufficiently efficient to completely remove target compounds. PFHxS and short-chain PFAAs were predominant in surface water samples, suggesting that they are being increasingly produced and applied due to regulations on PFOS and PFOA. Western inflowing rivers, such as the Paihe and Naifei Rivers, were the primary import routes of the PFAAs to Lake Chaohu. Additionally, the contamination levels of PFAAs in the western area of Lake Chaohu were generally higher than those in other areas, suggesting the remarkable effects of human activities on the residues of PFAAs in the aquatic environment. The high bioaccumulation potential in fish species was observed for PFDA, PFOS and PFNA. Further work is needed to study the bioaccumulation of PFAAs in organisms at different trophic levels in Lake Chaohu.

## Figures and Tables

**Table 1:** Estimated daily flux of perfluoroalkyl substances (PFAAs) in the effluent from sewage treatment plants (STPs) to Lake Chaohu.

STP	Flow (m^3^ day^−1^)	Concentrations (ng L^−1^)		Flux (g)	
		ΣShort-Chain PFASs	ΣLong-Chain PFASs	ΣShort-Chain PFASs	ΣLong-Chain PFASs
STP1	180,000	28.4	8.9	5.11	1.6
STP2	300,000	23.3	20.6	6.98	6.19
STP3	200,000	15.8	14.6	3.16	2.91
STP4	110,000	8.95	8.01	0.98	0.88
STP5	600,000	14.2	112	8.5	67.1
STP6	50,000	12	171	0.6	8.56
STP7	600,000	179	36.4	107	21.8
Sum.	2,040,000				

**Table 2:** Concentrations and frequencies of occurrence of 14 PFAAs in water samples of Lake Chaohu in 2015.

Analytes	Inflowing Rivers				Lake Sites			
	Detection Frequency (%)	Range	Median	Mean	Detection Frequency (%)	Range	Median	Mean
PFBA	100	15.2–52.2	28.3	31.1	100	12.7–46.2	30.7	29.5
PFPeA	100	11.1–67.5	41.5	37.7	100	9.06–236	44.5	57.5
PFHxA	100	2.9–16.9	3.86	7.26	100	2.42–6.55	4.46	4.73
PFHpA	89	ND–5.21	1.92	2.05	100	0.92–5.69	2.5	2.71
PFOA	100	8.68–25.4	16.9	16.9	100	17.1–33.3	20.82	21.6
PFNA	89	ND–2.29	1.15	1.15	100	0.64–3	1.3	1.53
PFDA	100	0.64–2.29	1.43	1.50	100	0.66–3.11	1.77	1.76
PFUnDA	56	ND– <LOQ	LOQ	LOQ	100	ND–7.07	ND	7.07
PFDoDA	56	ND– <LOQ	LOQ	LOQ	100	ND–9.95	ND	9.95
PFTriDA	33	ND– <LOQ	ND	LOQ	71	ND–18	ND	15
PFTDA	33	ND– <LOQ	ND	LOQ	79	ND–2.85	ND	2.14
PFBS	100	1.66–710	5.9	83.2	100	4.39–27	7.92	10.2
PFHXS	100	0.77–1866	4.89	211	100	3.44–168	18.50	32
PFOS	100	1.38–49.4	2.79	9.42	100	2.37–6.81	4.33	4.29

**Table 3:** Concentrations of PFAAs in water from Lake Chaohu and comparison with values reported in the literature.

Compound	Lake Chaohu, China (This Study)	Lake Chaohu, China [33]	Lake Taihu, China [50]	Bohai Sea, China [52]	Rivers, Swedish [53]	Rivers, Japan [51]
PFBA	12.7–46.2	0.31–6.77	ND–4.06	ND–2.9	0.47–3.7	ND–18
PFPA	9.06–236	0.03–8.12	ND–6.08	ND–7.91		ND–16
PFHxA	2.42–6.55	0.19–15.9	ND–22.2	ND–17.4	0.51–4.2	ND–16000
PFHpA	0.92–5.69	0.14–1.47	1.28–4.53	ND–1.46	0.36–1.7	ND–27
PFOA	17.1–33.3	1.32–23.5	2.15–73.9	ND–83.4	0.21–4.2	ND–360
PFNA	0.64–2.69	0.05–1.74	0.55–5.04	ND–0.53	0.09–5.8	ND–39
PFDA	0.66–3.11	0.02–0.7	ND–2.93	ND–0.93	0.02–4.4	ND–47
PFUnDA	ND–7.07	ND–0.12	ND–3.27	ND–1.4	0.02–1.8	ND–39
PFDoDA	ND–9.95		ND–0.89	ND–0.46	0.02–0.82	ND–4.1
PFTriDA	ND–18			ND		
PFTDA	ND–2.85			ND–0.21	0.09–1.5	
PFBS	4.39–27	0.03–6.14		ND–1.46	0.03–19	ND–49
PFHxS	3.44–168	0.01–0.96	ND–6.92	ND–0.28	0.05–18	ND–8.4
PFOS	2.37–6.81	ND–0.82	ND–10.5	ND–6.8	0.04–6.9	ND–97

**Table 4:** Concentrations (ng g^−1^), bioaccumulation factors (L kg^−1^) and log bioaccumulation factor (BAF) values for 14 PFAAs and fish samples from Lake Chaohu in 2015.

Analytes	Detection Frequency (%)	Concentrations (ng/g DW)		BAF		logBAF	logBAF [11]	logBAF [54]	logBAF [55]
		Min–Max	Mean	Min–Max	Mean				
PFBA	100	1.42–19.3	5.12	48.2–655	174	2.24		0.95–3.58	
PFPeA	100	0.64–12.4	3.18	11.4–128	55.3	1.74		3.53–3.94	
PFHxA	46	0.12–4.76	1.02	25.2–1007	469	2.67			
PFHpA	54	0.12–4.88	1.03	52.3–1801	706	2.85			
PFOA	100	0.26–4.17	1.34	12.1–193	61.9	1.79	0.99–1.94	2.91	1.32–2.08
PFNA	85	1.19–5.83	2.64	778–3813	2043	3.31	1.69–2.97		2.15–3.53
PFDA	100	0.33–13.9	5.6	186–7867	3183	3.5	1.48–3.75	4.3	2.20–3.98
PFUnDA	100	1.09–10.1	4.83	155–1427	684	2.83	2.50–4.17		2.79–4.45
PFDoDA	100	0.33–2.78	1.49	39.4–279	150	2.17	2.89–4.06		0.04–1.23
PFTriDA	92	0.44–2.78	1.23	29.2–186	93.3	1.97			
PFTDA	85	0.14–1.81	0.49	102–844	289	2.46			
PFBS	46	0.60–4.05	1.11	58.5–398	235	2.37			
PFHXS	46	0.14–1.79	0.41	4.33–55.7	27.9	1.45			
PFOS	100	0.44–50.6	9.73	102–11,794	2267	3.35	2.23–3.77	3.51–5.02	2.26–3.58

## Supplementary Materials

The following are available online at https://www.mdpi.com/1660-4601/16/10/1692/s1.
