# Supplementary material for: Occurrence, Removal and Bioaccumulation of Perfluoroalkyl Substances in Lake Chaohu, China
Source: Int J Environ Res Public Health. 2019 May 14;16(10):1692. doi: 10.3390/ijerph16101692 (PMC6572331; doi:10.3390/ijerph16101692)
Supplement: Supplementary file 1 [file ijerph-16-01692-s001.pdf]

Table S1. Optimized MRM parameters for the quantification of PFAAs.

Compounds	Precursor Ion	SRM$_1$ [a]	Cone voltage (V)	Collision voltage 1 (eV)	SRM$_2$ [b]	Collision voltage 2 (eV)
**Perfluoroalkyl Substances**						
Perfluorobutyric acid(PFBA)	213	213	20	2		
Perfluoropentanoic acid (PFPA)	263	263	20	2	219	5
Perfluorohexanoic acid (PFHxA)	313	269	16	10	119	17
Perfluorohetanoic acid (PFHpA)	363	319	16	10	169	19
Perfluorooctanoic acid (PFOA)	413	219	16	17	369	10
Perfluorononanoic acid (PFNA)	463	219	16	17	169	19
Perfluorodecanoic acid (PFDA)	513	469	16	10	219	17
Perfluoroundecanoic acid (PFUnDA)	563	519	16	10	319	17
Perfluorododecanoic acid (PFDoDA)	613	569	16	10	169	22
Perfluorotridecanoic acid (PFTriDA)	663	619	16	10	169	14
Perfluorotetradecanoic acid (PFTDA)	713	669	16	10	169	5
Perfluorobutane Sulfonate (PFBuS)	299	99	40	31	80	30
Perfluorohexane Sulfonate (PFHxS)	399	99	45	31	80	30
Perfluorooctane sulfonic acid (PFOS)	499	80	45	40	99	41
**Internal Standards**						
Perfluoro-n-[1,2,3,4-$^{13}C_4$] butanoic acid ($^{13}C_4$-PFBA)	217	172	20	2		
Perfluoro-n-[1,2-$^{13}C_2$] hexanoic acid ($^{13}C_2$-PFHxA)	315	270	12	9	119	5
Perfluoro-n-[1,2,3,4-$^{13}C_4$] octanoic acid ($^{13}C_4$-PFOA)	421	376	16	10	169	13
Perfluoro-n-[1,2-$^{13}C_2$] decanoic acid ($^{13}C_2$-PFDA)	515	470	15	11	270	12
Perfluoro-n-[1,2-$^{13}C_2$] dodecanoic acid ($^{13}C_2$-PFDoDA)	615	570	18	12		
Perfluoro-1-hexane[$^{18}O_2$] sulfonate ($^{18}O_2$-PFHxS)	403	103	40	30	84	37
Perfluoro-1-[1,2,3,4-$^{13}C_4$] octanesulfonate ($^{13}C_4$-PFOS)	503	80	60	39	99	41

[a] SRM$_1$ = selected product ion for quantification; [b] SRM$_2$ = selected product ion for qualification.

Table S2. Relative recoveries obtained for spiked water and fish samples

PFASs	Internal Standard	Surface Water [a]		Influent Water [a]		Effluent Water [a]		Fish [b]	
		Rec. ± RSD	LOQ (ng L^{-1})	Rec. ± RSD	LOQ (ng L^{-1})	Rec. ± RSD	LOQ (ng L^{-1})	Rec. ± RSD	LOQ (ng g^{-1})
PFBA	MPFBA	67 ± 5	0.01	92 ± 10	0.55	105 ± 3	0.24	64 ± 16	0.50
PFPA	MPFBA	60 ± 1	0.02	112 ± 6	0.60	106 ± 3	0.30	72 ± 15	0.40
PFHxA	MPFHxA	74 ± 10	0.02	118 ± 1	0.07	94 ± 8	0.03	67 ± 10	0.05
PFHpA	MPFHxA	87 ± 1	0.01	98 ± 5	0.04	105 ± 3	0.02	74 ± 10	0.05
PFOA	MPFOA	107 ± 6	0.01	120 ± 2	0.04	102 ± 13	0.03	68 ± 9	0.10
PFNA	MPFOA	92 ± 6	0.02	108 ± 4	0.11	99 ± 12	0.12	76 ± 16	0.40
PFDA	MPFDA	86 ± 3	0.03	92 ± 2	0.08	87 ± 5	0.13	77 ± 12	0.15
PFUnDA	MPFDA	88 ± 4	0.02	74 ± 14	0.06	78 ± 5	0.10	79 ± 10	0.30
PFDoDA	MPFDoA	70 ± 7	0.02	78 ± 7	0.05	64 ± 7	0.06	67 ± 13	0.10
PFTriDA	MPFDoA	70 ± 11	0.02	56 ± 14	0.18	52 ± 13	0.07	78 ± 15	0.12
PFTDA	MPFDoA	71 ± 16	0.04	61 ± 9	0.18	83 ± 15	0.09	70 ± 14	0.05
PFBuS	MPFHxS	58 ± 2	0.03	97 ± 7	0.06	103 ± 3	0.04	109 ± 13	0.10
PFHxS	MPFHxS	87 ± 5	0.02	99 ± 5	0.05	104 ± 3	0.02	64 ± 13	0.05
PFOS	MPFOS	77 ± 5	0.02	100 ± 7	0.12	105 ± 11	0.11	71 ± 14	0.12

[a]: spiked at a concentration of 10 ng L^{-1}; [b]: spiked at a concentration of 10 ng g^{-1}

Table S3. Occurrence and distribution of PFAAs in Lake Chaohu in October 2015 (ng L^{-1})

Compounds	S1	S2	S3	S4	S5	S6	S7	S8	S9	S10	S11	S12	S13	S14
PFBA	34.5 ± 1.82	34.2 ± 3.82	26.4 ± 5.16	32.2 ± 6.42	34.1 ± 0.87	29.8 ± 2.41	23.3 ± 0.15	12.7 ± 1.84	31.7 ± 1.91	33.1 ± 4.65	28.9 ± 2.56	26.7 ± 2.98	46.2 ± 6.71	18.9 ± 2.32
PFPA	19.0 ± 1.97	68.8 ± 8.05	44.4 ± 7.88	37.7 ± 4.03	79.0 ± 11.88	66.1 ± 5.95	9.06 ± 0.04	24.8 ± 1.98	51.1 ± 2.66	37.3 ± 0.94	46.8 ± 2.97	44.5 ± 4.68	236 ± 31.21	41.6 ± 7.38
PFHxA	6.48 ± 0.29	5.49 ± 0.35	3.91 ± 0.24	5.99 ± 0.29	5.81 ± 0.15	4.13 ± 0.14	4.09 ± 0.81	2.42 ± 0.06	4.76 ± 0.13	3.76 ± 0.26	3.87 ± 0.63	4.5 ± 0.45	6.55 ± 0.42	4.41 ± 0.49
PFHpA	2.39 ± 0.19	2.48 ± 0.33	2.19 ± 0.52	2.55 ± 0.32	1.99 ± 0.31	3.19 ± 0.41	3.10 ± 0.92	0.92 ± 0.14	3.51 ± 0.34	3.11 ± 0.13	2.56 ± 0.12	2.08 ± 0.04	5.69 ± 0.47	2.22 ± 0.17
PFOA	18.2 ± 0.41	17.3 ± 0.3	23.4 ± 1.53	21.2 ± 0.35	21.3 ± 1.96	20.5 ± 0.59	19.8 ± 0.55	17.1 ± 0.68	21.7 ± 0.68	24.2 ± 0.7	20.3 ± 2.35	23.2 ± 0.87	33.3 ± 3.61	20.0 ± 2.79
PFNA	1.33 ± 0.24	1.12 ± 0.03	0.83 ± 0.07	0.93 ± 0.60	0.64 ± 0.28	2.41 ± 0.07	1.95 ± 0.36	0.66 ± 0.29	3.0 ± 0.45	2.69 ± 0.24	2.07 ± 0.26	1.27 ± 0.72	1.48 ± 0.24	1.06 ± 0.39
PFDA	1.75 ± 0.07	1.57 ± 0.04	1.12 ± 0.16	1.64 ± 0.18	1.79 ± 0.16	1.92 ± 0.76	1.83 ± 0.53	0.66 ± 0.05	3.11 ± 0.21	2.57 ± 0.25	1.85 ± 0.46	1.04 ± 0.04	2.78 ± 0.54	0.97 ± 0.18
PFUnDA	<LOQ	<LOQ	<LOQ	<LOQ	<LOQ	<LOQ	<LOQ	<LOQ	7.07 ± 0.49	<LOQ	<LOQ	<LOQ	<LOQ	<LOQ
PFDoDA	<LOQ	<LOQ	ND	<LOQ	<LOQ	<LOQ	<LOQ	ND	9.95 ± 0.55	<LOQ	<LOQ	<LOQ	ND	ND
PFTriDA	<LOQ	ND	<LOQ	<LOQ	ND	<LOQ	<LOQ	<LOQ	18.0 ± 2.08	12.0 ± 0.54	<LOQ	<LOQ	ND	<LOQ
PFTDA	<LOQ	ND	ND	<LOQ	<LOQ	<LOQ	<LOQ	2.26 ± 0.68	2.79 ± 0.99	<LOQ	0.65 ± 0.09	<LOQ	2.85 ± 0.40	<LOQ
PFBuS	11.7 ± 0.71	15.4 ± 0.94	5.89 ± 0.28	15.0 ± 0.77	27.0 ± 1.98	6.52 ± 0.54	6.02 ± 0.68	4.39 ± 0.05	8.37 ± 0.3	6.62 ± 0.23	5.78 ± 0.09	7.92 ± 2.19	13.9 ± 0.29	7.92 ± 1.12
PFHxS	33.9 ± 1.95	56.9 ± 4.43	7.21 ± 2.72	45.2 ± 0.92	168 ± 12.8	8.55 ± 0.16	5.11 ± 0.18	3.86 ± 0.03	6.45 ± 0.41	6.17 ± 0.2	3.44 ± 0.41	32.0 ± 4.84	42.9 ± 1.41	28.4 ± 9.43
PFOS	4.67 ± 0.25	5.56 ± 0.58	2.85 ± 0.48	5.5 ± 0.19	6.81 ± 0.46	2.92 ± 0.32	2.85 ± 0.24	2.37 ± 0.07	5.18 ± 0.1	3.5 ± 0.21	2.86 ± 0.15	4.97 ± 0.97	5.98 ± 0.27	4.0 ± 0.84
ΣPFASs	134	209	118	168	347	146	77.1	72.1	177	135	119	148	397	129

Table S4. Components of short- and long- chain PFAAs in water from inflowing rivers

Rivers	ΣPFAAs (ng/L)	Ratios (%)			
		**Short-PFCAs**	**Long-PFCAs**	**Short-PFSAs**	**Long-PFSAs**
ZGH	120	68.92	19.18	10.00	1.89
BSTH	91.5	53.68	32.48	10.67	3.17
TYH	102	73.38	18.47	6.28	1.87
HBH	135	86.61	10.56	1.81	1.02
NFH	166	78.77	10.49	4.76	5.98
ZH	70.1	46.48	29.94	19.71	3.88
SQH	82.6	53.53	29.48	13.60	3.38
SWLH	98.7	70.24	11.67	6.56	11.53
PH	2743	3.67	0.60	93.92	1.80
YXH	73.5	64.39	19.96	13.10	2.55

Table S5. Components of short- and long- chain PFAAs in water from Lake Chaohu.

Sites	ΣPFAAs (ng/L)	Ratios (%)			
		Short-PFCAs	Long-PFCAs	Short-PFSAs	Long-PFSAs
S1	134	44.79	17.67	8.75	28.79
S2	209	51.93	10.75	7.39	29.94
S3	118	63.22	23.29	4.98	8.51
S4	168	45.19	15.65	8.93	30.23
S5	347	34.27	7.41	7.79	50.53
S6	146	68.48	19.19	4.47	7.86
S7	77.1	47.24	34.63	7.81	10.33
S8	72.1	55.34	29.94	6.09	8.64
S9	177	49.56	39.11	4.74	6.59
S10	135	54.96	32.97	4.91	7.17
S11	119	66.84	23.02	4.85	5.29
S12	148	51.09	18.62	5.35	24.95
S13	397	72.60	11.60	3.49	12.31
S14	129	50.10	18.71	6.12	25.07
